# *Varroa destructor* from the Laboratory to the Field: Control, Biocontrol and IPM Perspectives—A Review

**DOI:** 10.3390/insects12090800

**Published:** 2021-09-07

**Authors:** Caroline Vilarem, Vincent Piou, Fanny Vogelweith, Angélique Vétillard

**Affiliations:** 1 Laboratoire Evolution et Diversité Biologique, UMR5174, CNRS-Université de Toulouse III-IRD, INU Jean-François Champollion, Université Paul Sabatier, 31077 Toulouse, France; caroline.vilarem@m2i-group.fr (C.V.); vincentpiou@live.fr (V.P.); 2 M2i Biocontrol–Entreprise SAS, 46140 Parnac, France; fanny.vogelweith@m2i-group.fr

**Keywords:** *Varroa destructor*, honeybees, integrated pest management, biocontrol, holistic approach

## Abstract

**Simple Summary:**

*Varroa destructor* is a parasitic organism feeding and living among honeybees. It transmits viruses like the Deformed Wing Virus which can lead to the decline and death of the colony. Many treatments have been developed over the years like formamidine amitraz, pyrethroid tau-fluvalinate, organophosphate coumaphos or even acids like formic and oxalic to control the spread of the mite. However, none of this solution provides long-term sustainability for honeybees and no resistance from *V. destructor*. Therefore, the development of alternative tools remains open. This will require the combination of both laboratory and field results through an integrative approach based on the identification of *V. destructor* health biomarkers. Here we review what has been done and what can be done from the laboratory to the field against the parasitic pressure held on honeybees.

**Abstract:**

*Varroa destructor* is a real challenger for beekeepers and scientists: fragile out of the hive, tenacious inside a bee colony. From all the research done on the topic, we have learned that a better understanding of this organism in its relationship with the bee but also for itself is necessary. Its biology relies mostly on semiochemicals for reproduction, nutrition, or orientation. Many treatments have been developed over the years based on hard or soft acaricides or even on biocontrol techniques. To date, no real sustainable solution exists to reduce the pressure of the mite without creating resistances or harming honeybees. Consequently, the development of alternative disruptive tools against the parasitic life cycle remains open. It requires the combination of both laboratory and field results through a holistic approach based on health biomarkers. Here, we advocate for a more integrative vision of *V. destructor* research, where in vitro and field studies are more systematically compared and compiled. Therefore, after a brief state-of-the-art about the mite’s life cycle, we discuss what has been done and what can be done from the laboratory to the field against *V. destructor* through an integrative approach.

## 1. Introduction

While honeybees forage, nurse, reproduce, eat, or communicate in an already critical unhealthy environment with pesticides [1], climate change [2] or habitat loss [3], parasites take their chance too [4]. One of them is *Varroa destructor*, a world major threat against bees [5,6]. After a shift from its original host the Asian bee *Apis cerana* to the Western honeybee *Apis mellifera*, it rapidly spread in the 1970s in Europe and in the 1980s in America [5]. It is now observed in both managed and wild *A. mellifera* [7]. Due to a shorter coevolution time between *A. mellifera* and *V. destructor* [8] as well as fundamental biological differences, the Western honeybee is far more impacted by the mite than the original host [5,9,10,11].

Why is this ecto-parasite such a threat for *A. mellifera* worldwide? It appears that *V. destructor* is tightly connected to several viruses and especially the DWV (Deformed Wing Virus) with its diverse variants DWV-A, DWV-B originally known as VDV-1 and DWV-C [12,13,14]. This RNA virus is responsible for wing malformations in bees, causing flight incapacities, thus a lack in food collection for the colony but also a threat to pollination [15,16]. Assuming the transmission of the virus occurs at the adult stage, no visible symptoms are reported [17] but a shortened lifespan is described [18]. The infection at the larval stage (emphasised through *V. destructor*) induces damage like shortened abdomens, reduced brood nursing and learning deficits [19,20]. From a host-parasite perspective, interactions have to be considered not as a duo but as a triangle, highlighting the virus quasispecies which can spread between bees and acari [17,21,22,23,24,25,26].

Fifty years of intense research about *V. destructor* support an impressive amount of knowledge in order to deal with practical issues: how to reduce the impact of the mite on honeybee populations [27]? It is true that current control methods efficiency for the acari are still debated. Hard chemicals like pyrethroids, formamidine, organophosphate, neonicotinoid, or sulfoximines were or are still used in the field. However, their negative impact on honeybees’ cognition is now widely identified and the resistance developed by the mite is part of current knowledge [28,29]. Other solutions were explored too, like drone removal, brood interruption, or breeding programs [30,31]. Soft acaricides such as thymol, hop leaves or acids seem promising for some [32,33] and already trouble making for others [34,35].

As urged by the integrated pest management program (IPM), a more integrative view is compulsory. The goal is not anymore to kill each and every one of them but rather to reduce their impact without harming honeybees and other wild species around. New strategies based on IPM and biocontrol are passionately studied all over the world. Currently, none is adequate to reduce the adverse impact of mites on bees. It is imperative to develop a holistic approach that focuses on the complete understanding of *V. destructor* biology and its tight relationship with its host. Health biomarkers should be determined for the mite and would help to evaluate at sub-lethal level on a long-term period the impact of molecules or biotechnics. This integrative approach involves in silico, in vivo, semi-field and field scales. Our review aims to discuss the latest ideas about control, IPM and biocontrol for *A. mellifera* against *V. destructor* from the laboratory to the field in realistic reproductible and applicable conditions. 

## 2. Know Your Challenger, *Varroa destructor*

### 2.1. The Ecto-Parasite’s Life Cycle

**Figure 1:**
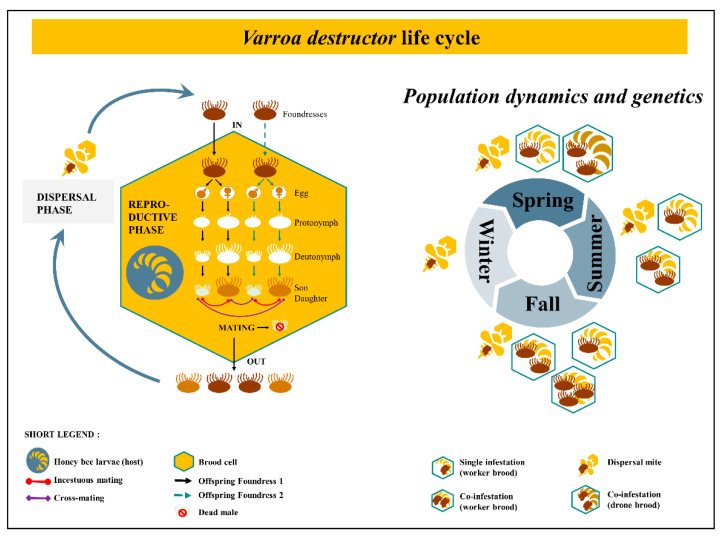
*Varroa destructor* life cycle and its population dynamics throughout the year. On the left, the two phases of the ecto-parasite cycle. The black line is in case of single infestation, the dashed line is in addition of the black one, thus in case of co-infestation in a brood cell. Inside the brood cell, the foundress laid eggs which go through several steps from protonymph to deutonymph until becoming a new daughter and son. On the right, the pattern of infestation over time. The balance between the number of brood available and the number of ecto-parasites in the colony influence the genetic diversity. *Varroa destructor* mating biology leads to intense inbreeding with no exchange among lineages in the population while a single foundress invades a brood cell in spring. Co-infestation happens in drone brood cell early in the year. However, cross-mating events occur around summer and fall with two or more foundresses co-infesting the same cell resulting in more heterozygous loci [54]. Contrary to assumptions, the ecto-parasite is genetically diversified with a dynamic structure over time [48].

A great dynamic for *V. destructor* research has enlightened the domain, giving us new comprehension about the parasite’s interaction with its host. The mite’s cycle is composed of two distinct phases. The dispersal phase, previously called phoretic phase [36,37] refers to the periods during which the mite feeds, travels on adult honeybees, allows its spermatophores to mature and activates ovaries [38]. The reproductive phase refers to the part of the cycle which takes place within a brood cell, where the parasite feeds on the bee throughout its development (from larvae up to imago). Once the female mite invades a worker brood cell holding a larval bee from 15 to 20 h before capping [39], within a few hours the oogenesis begins [40] and 60 h post cell capping the first egg is laid [41]. New borns feed from the hole (100 μm) pierced by the mother’s chelicerae in the pupa [42,43]. The feeding pit on bee pupae stands open due to anticoagulants from the ecto-parasite saliva and suppression of healing processes [37,44,45]. The first born is always a male (haploid) while the following eggs laid are females (diploid). New borns go through several steps of development, from protonymphs to deutonymphs that molt into adult mites (Figure 1). Once they have reached the adult stage (9–10 days after cell capping for the first born female) and until emergence, mating occurs between brother and sisters in case of single infestation [46,47]. Cross-mating are possible when two or more foundresses invade the same cell [48]. Multiple mating events happen within the cell, until the bee reaches its imaginal stages and emerges. At this point, the male dies and the newly fecund females climb on this new born honeybee, along with their mother, to leave the cell as well. A new generation is born, and the life cycle of the mite goes on with the dispersal (ex phoretic) phase. Based on artificial experiments and anatomical observations, a female would be able to do a series of seven reproductive phases before it dies [49,50]. A third state is viable during the lifetime of *V. destructor*. Out of the brood cell or off the honeybee body, the female acari moves freely on the surface of the comb [51] and therefore can survive a few hours without a host. 

The population dynamics, structure and genetics of the mite are crucial to understand the spreading process as well as their adaptations and resistances [52,53]. *Varroa destructor* mating biology leads to intense inbreeding with no exchange among lineages in the population while a single foundress invades a brood cell. However, cross-mating events occur with two or more foundresses co-infesting the same cell resulting in more heterozygous loci [54]. In temperate climate like Europe, during spring at the beginning of the season, single infestations happen due to excess of bee brood compared to the number of ecto-parasites. During summer and fall, the number of available brood cell decreases while *V. destructor* population increases, therefore co-infestations occur intensely (Figure 1). Contrary to initial assumptions, the ecto-parasite is thus, genetically diversified with a dynamic structure over time as it varies according to brood availability in the colony [48]. In addition, genetically different incomers arrive with workers drifting from surrounding colonies [55,56]. The host seasonal timeline is a key parameter for genetically based resistance toward acaricides [57]. In fact, temporal rotation between incestuous mating and cross-mating are critical in the failure of controlling *V. destructor*. Inbreeding increases the frequency of homozygotes with resistant alleles to molecules even before any cure. According to genetic population dynamics and structure, treatment should occur during cross-mating period [48].

Besides allowing genetic diversity, the cycle and more specifically the synchronicity between *V. destructor* and its host would not be possible without a chemical communication network. 

### 2.2. Eavesdropping and Hijacking the Hive: V. destructor, the Perfect Scrounger

Pheromones are known to be a key factor in colony communication, as they maintain the unity and plasticity of the honeybee society [58,59]. In the present work, we will only focus on some semiochemicals thought to be involved in the host–parasite interactions with *V. destructor*, for a better understanding of the challenger biology.

ORIENTATION—Many olfactory signals from adults, brood, or colony matrices have elicited behavioural responses from the mite. For instance, specific blends of fatty acid esters from old bee larvae [60,61] or aliphatic alcohols and aldehydes from cocoons [62] were shown to trigger the arrest or even the attraction of female acari in laboratory conditions. Brood food holds 2-hydroxyhexanoic acid, a volatile blend which also appeals dispersive mites [59,63]. Conversely, the mite is blocked by the ω-functionalised fatty acids from royal jelly [64] and the larger amount of methyl oleate in royal cells [65], preventing the parasitisation of queen brood. The ecto-parasite is also blocked and pushed away by two components of the Nasonov pheromone, geraniol and nerolic acid [66] as well as (Z)-8-heptadecene [67]. It turns out that foragers emit more of this semiochemicals and the (Z)-8-heptadecene than nurses. Therefore, it partially explains why dispersal mites are able to choose nurses over foragers [68,69].

Even *V. destructor* movements across the colony are primarily oriented through volatile compounds catched by their chemosensory organs located in forelegs [70]. Nganso et al., (2020) [71] demonstrated by mechanically blocking them with nail polish that the mite has lower ability to select and identify a suitable host to reproduce.

REPRODUCTION—In the dark environment of the capped cell, the male acari has to recognise and mate specifically with the mature unmated females. The female actually emits a volatile sexual pheromone, composed of oleic acid, palmitic acid, stearic acid and their ethyl esters which attracts the male through its tarsal sensory pits and triggers its courtship behaviour [72,73]. The youngest daughter seems to be always the favourite choice of the male because the emission of the sexual pheromone is stronger in young and reduced in older females [72]. In the same way, once the mite is inside a capped brood cell, it is the shift in the fatty acid ester compound produced by the bee pupa (a decrease of ethyl esters and an increase of methyl esters) that initiates the reproduction and egg laying through vitellogenin induction [74,75]. Even the sex determination seems to be driven by bee pheromones as Garrido and Rosenkrank [76] showed. In that case, fatty acid esters volatile signal triggers a male egg laying by the foundress. Supporting the hypothesis of a complex chemical environment ruling *V. destructor* behaviour, Frey et al. [75] demonstrated that artificially inserted mites with methyl esters compound stop reproduction. Plettner et al. [59] hypothesised that this signal alone could indicate to the mite that the bee pupa development is too advanced for the offspring to reach adulthood before the honeybee emergence.

CAMOUFLAGE—*Varroa destructor* has not only chemosensing skills but also undertakes chemical mimicry to influence its host [37,77]. The mite avoids recognition by worker bees that would clean the infested cell or clear their nestmate body carrying acari. As a result, *V. destructor* hijacks host communication signals to bypass exposure [73]. Indeed, the ecto-parasite and honeybees, whatever the developmental stage, share a typical pattern of alkane like pentacosane or heptacosane [78]. In fact, Kather et al., (2015) [79] demonstrated that *V. destructor* is unable to biosynthesise host compounds itself (e.g., chemical mimicry) but instead requires direct access to the cuticle for odour passive acquisition (e.g., chemical camouflage) [80]. This camouflage mechanism is extremely plastic and takes only up to 9 h after host shifting during the reproductive but also dispersal phases.

According to the period and the hive environment, the behaviour of *V. destructor* is quite different and extremely adaptive [81]. At low mites abundance in the colony, acari seem to prefer nurse bees over foragers or new born bees [82], based on differential pheromonal signatures between nurses and foragers. Yet, the mite can passively modify the hydrocarbon cuticle of its host according to colony infestation levels. At high infestation levels, nurses and foragers, less discernible by their different cuticular hydrocarbons, are equally appealing to *V. destructor*, thus promoting the exploration of new bee colonies [83]. 

This natural drifting of mites between colonies is one of the factors which increases the deleterious effect of the ecto-parasite on bee populations, especially through viral transmission. The biology of the ecto-parasite is indeed not complete without the study of its viruses like the Deformed Wing Virus.

### 2.3. Varroa destructor and DWV—A Cut-Throat Association to Bees

The host–parasite relationship between honeybees and the ecto-parasite is more complex than a duo and takes a third dimension associated with viruses like the DWV. This multipartite interaction is not fully understood. The ecto-parasite plays a central role in the recent expansion of the DWV [16]. It turned this originally benign virus, showing relatively asymptomatic infection, to a highly virulent virus associated with disease symptoms and colony losses [84,85,86]. 

*Varroa destructor* spread also made an impact on the virus genetics and resulted in selection of virulent variants of DWV [87,88,89]. A particular DWV variant, namely DWV-B, infects the gut epithelium and salivary glands of the ecto-parasite [24], establishing it as a biological virus vector since it multiplies inside the vector [17,25,26]. Furthermore, *V. destructor* enhances the development of DWV level in the European honeybee through a saliva protein [22], the same that is toxic for *A. cerana* worker larvae. Equally important, the predator–prey theory developed by Volterra seems to apply in this triangle connection [90]. The haemolymph punction, that could be associated with the parasitisation, destabilises the immune system, accelerates the immunosuppression and causes explosive replication of the DWV in infected bees [91,92]. 

What are the effects of DWV on *V. destructor* physiology? Few studies explored the direct impact of the virus on the mite for itself. The main reason is due to the recent advances which showed that *V. destructor* is not only a carrier but as well an amplificator of the virus. One of these studies localised the DWV in the ecto-parasite synganglion questioning the host manipulation hypothesis where the mite behaviour could be altered to favour the transmission of the virus [93,94]. More research would help to identify health biomarkers of *V. destructor* that can be influenced by the DWV.

All these key parameters in the understanding of the host/parasite interactions could be as many promising therapeutic targets to control *V. destructor.*

## 3. *Varroa destructor* Chemical and Semi-Chemical Control Methods 

### 3.1. Hard Acaricides

Synthetic acaricides were first used in the 1970s to effectively fight against the acari [95,96]. They were easy to use, cheap and effective [5]. Different types were released in the market followed by several research works describing their impact on the mite but also collateral impacts on bees. The formamidine amitraz, pyrethroid tau-fluvalinate and organophosphate coumaphos are the three active ingredients and major hard acaricides used against *V. destructor*. Their mode of action is now well described in the literature. For example, formamidine amitraz holds a toxic effect by interacting with the octopamine receptors involved in insects’ nervous system [97]. It inhibits neurotransmission leading to paralysis [98]. As for tau-fluvalinate, it changes kinetics of the voltage-gated sodium channel. The axonal membrane permanently depolarised induces the paralysis of the mite [31,99]. In addition, other pyrethroids are known to increase the transcription level of antimicrobial peptides and hymenoptaecin (an antibacterial polypeptide) in bees [100,101,102] leading to a modified physiology. The last major acaricide, coumaphos, acts as an acetylcholinesterase inhibitor and impedes nerve signalling [103,104]. 

Due to their modes of action and their impact on gene expression, several works showed that commercial acaricides harm honeybees lethally and sub-lethally. While a few studies do not detect any detrimental effect [105], some have shown olfactory memory impairment [106] or locomotor deficits [28] associated with the presence of acaricide. Another major issue is due to lipophilic properties of these molecules which facilitate their accumulation in bee products especially in wax, thus surexposing honeybees and *V. destructor* to the substance [107,108,109]. Another drawback to keep in mind is the inability of hard acaricides to reach capped brood, killing the ecto-parasite only out of the reproductive state. 

One more exploratory option resides in lithium chloride. Ziegelmann et al. [110] investigated its acaricidal impact by feeding honeybees and analysed their lifespan. This molecule, not yet on the market, showed efficient results against dispersal mites through oral and contact delivery methods [111,112]. However, the question arose about honeybee products potential contamination [113]. Further research is still needed to determine long-term and sub-lethal effects on bees in the laboratory as well as in the field.

The off-target effects, along with the increasing resistances of mites, pushed for new ways of pest control [57] like soft acaricides. 

### 3.2. Soft Acaricides 

Many molecules from natural origins were considered as alternatives to synthetic chemical treatments. Thymol, formic acid or oxalic acid have been stressed since the 1970s–1980s as efficient treatments against the ecto-parasite [114]. Yet, the ‘perfect’ solution does not exist, and new molecules are tested each year in the hope to come up with a long-term answer. Some of these molecules have been used for decades without a clear understanding of their mode of actions on both the honeybee and the acari. This is for instance the case of formic acid. 

FORMIC ACID—Formic acid holds a great potential since it is the only molecule, so far, able to reach both dispersal and reproductive mites inside brood cells [115]. When used as a treatment, formic acid seems to interfere with the cellular respiratory chain, more precisely the cytochrome C oxidase. It inhibits the oxidative phosphorylation, thus impacting the mitochondrial energy metabolism [116]. Genath et al., (2020) [117] studied the transcriptome of *A. mellifera* and *V. destructor* after topical formic acid treatment and highlighted a difference in detoxification capacity between the host and the acari. Their work supports the hypothesis of interference with cellular respiration through the modified expression of several genes like cytochrome P450 suggesting a stronger toxic selectivity toward the mite. To date, no resistance was detected in *V. destructor* [59,73]. Hansen et Guldborg (1988) [118] showed that the formic acid concentration increasing in honey after a treatment was not sufficient to be harmful and persistent in time [119,120]. Nevertheless, many parameters such as the delivery methods, the size of the hive, the position of the evaporator in the hive, the humidity and the temperature are known to greatly affect the treatment efficacy [121,122,123,124]. For example, high temperature combined with low ventilation in-hive may lead to higher brood toxicity and lower mite mortality due to quick evaporation rates [125]. In addition, several studies described some drawbacks like swarming, queen mortality, damaged young bees or reduction of sealed brood [123,126,127]. At sub-lethal doses, formic acid involved memory impairment for bees in the short and long term [106]. Still, this acid seems a good compromise to keep a reduced number of mites without drastic honeybees loss [123]. In addition to health risk for the user in case of incorrect use, real efficacy is known to variate throughout the world, from 39.8% of mite mortality in the USA [125] to 92% in Argentina [128].

OXALIC ACID—Oxalic acid is a natural acaricide in use since the 1980s against the mite. Again, this acid is a molecule naturally present in honey [119]. Due to its hydrophilic nature, oxalic acid is used as an acaricide treatment and does not lead to high residual concentration in wax [129,130,131,132]. It kills dispersive mites on honeybee body but cannot penetrate a brood cell, limiting its effects [130,133,134]. In field trials, Maggi et al., (2016) for instance, showed a miticide efficacy of 93.1%. Surprisingly its mode of action towards *V. destructor* is still unclear although it is most likely mechanical [73]. Sublimation method seems to cause crystallisation of the acid on the acari’s body, leading to the inability of the mite to adhere to any substrate [135]. The fact that *V. destructor* appears unable to detect this acid by olfaction [135] and the putative mechanical mode of action could reduce the chance of resistance from the mite [73]. No resistance was observed over 8 years of treatment in a recent bioassay [32]. Yet, caution should be taken as bacteria characterised from *V. destructor* microbiota were shown to express oxalotrophy. This gives them the ability to degrade oxalic acid in order to use it as their carbon source [136], thus conferring resistances to the carrying mites. Despite its use as an organic treatment, oxalic acid at high and sub-lethal doses can still be harmful to the bees. Severe and irreversible internal tissue damages [137,138] or disruption of the proteolytic activity of the cuticle were observed, impeding bees’ immunity [139]. Administration method is actually a key point and higher death rates were associated with oral exposure when compared to topical application [140]. Maggi et al. [32] suggested that the combination of glycerol with oxalic acid may prevent honeybees from oral ingestion, reducing deleterious effects, without reducing the efficacy of the acaricide treatment. Besides the effect on adult bees, experiments led on larval stages with spray application showed midgut damages as well [141]. Finally, tests on long-term effects on colonies characterised loss of brood, workers and sometimes queen according to the concentration used [130,142]. 

OTHERS—In addition to formic and oxalic acid, several other acids were screened and their effects against *V. destructor* were assessed. While citric and acetic acids were disappointing attempts [143,144], costic and oleic acids sound promising. The extraction of costic acid from *Dittichia viscosa* plant allowed field tests with a miticide effect of 80% without apparent bee mortality [145]. More precisely, alpha-costic acid was identified as the compound responsible of the acaricidal activity and was also shown to knock out the mite without killing it [146]. A later work even combined oxalic and costic acids to increase the compound efficiency against *V. destructor* [147]. Despite these promising results, sub-lethal effects on honeybees still need to be precisely explored in vitro and during field trials. 

As for oleic acid, it is involved in different biological functions and would constitute a key death pheromone eliciting hygienic behaviour in honeybees [148] and a sexual pheromone for *V. destructor* [72]. Regarding the latest, the authors discovered that a pest management solution through sexual confusion was possible in vitro and on semi-field trials [149]. However, even if oleic acid seems encouraging, it could be a tricky solution to put in use because of its omni-presence and its role in the regulation of diverse behaviours in bee colonies.

Besides acids, essential oils and derivatives were alternatives with good miticide effects [150]. Thymol is commercialised since the 1990s [151]. Derived from thyme oil, it is the most common essential oil-based product used against *V. destructor***.** Unfortunately, due to the accumulation in wax, scientists uncovered severe sub-lethal consequences for honeybees [152] like low larvae survival rate, delayed vitellogenin expression [34] or altered specific memory traces [153]. Thymol binds to dopamine receptors, which irritates honeybees and can modify the taste of honey [154,155]. Moreover, it triggers higher hygienic behaviour from bees [156]. Many other essential oils have been tested, and some were even commercialised. For instance, savoury or spearmint oils were investigated and showed acaricidal properties with low rate of honeybee mortality while dillsun induced higher death rates [157]. Menthol in sugar syrup displayed encouraging short-term results [150,158] whereas neem oil killed mites [159], but also increased brood mortality and reduced the worker’s walking activity [160].

Finally, another plant extract, relying on hop leaves, was shown to contain polyphenols with high miticide effect and low acute toxicity for bees [161,162]. An advantage of hop extracts comes from their antioxidant activity for honeybees, benefiting them if administered orally [33]. 

### 3.3. Years Outcome of Restricted Therapeutic Arsenal: Varroa destructor Biotype Disparities 

Adaptability strikes particularly in the original host of the mite, *Apis cerana*. Indeed, the long host–parasite coevolution has led to fitness optimality for both through natural selection process. It is well described in the literature that *V. destructor* females reproduce only in drone brood cells in *A. cerana* hive [163] thus, confining them in a restraint area and timeline. In addition, the Asian bees maintain efficient hygienic behaviour against the mite with infested brood removal, entombing or grooming a nestmate [164,165,166], thereby maintaining an equilibrium [9]. It is a different story with *A. mellifera* so far where the co-evolution is shorter than *A. cerana*. Nevertheless, several traits such as varroa sensitive hygiene (VSH) or suppressed mite reproduction (SMR) emerged and are still broadly studied [167,168,169]. In the meantime, chemical solutions were used to face this invader. Hard acaricides were largely described in the literature for causing resistance in the parasite due to long-term exposure to high doses of chemicals. In the colony, the mite population can be divided in three categories: susceptible, tolerant and resistant [170]. Mechanisms of resistance can take several forms such as behavioural acaricide avoidance, desensitisation through changes in the active site, diminished penetration or modulation of detoxification enzyme expression [171]. A majority of acaricides target crucial proteins from the nervous system leading to resistant mites through protein modifications.

**Figure 2:**
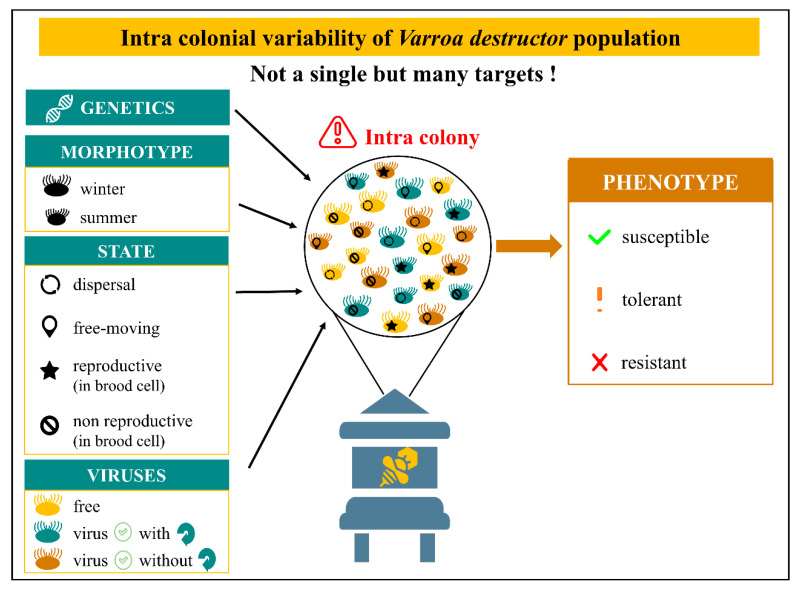
Intra colonial variability of *V. destructor* population. Genetics through several traits can influence the phenotype [168]. Mites differ from winter to summer with a dynamic morphotype ratio going from 20% of winter morphotype in summer to 20% of summer morphotype in winter [173,174]. In addition, *V. destructor* can be divided in four different states: dispersal ecto-parasite attached to honeybee’s body, comb mite freely moving inside the hive, reproductive acari confined in brood cell and non-reproductive mite confined in brood cell [30,172]. Virus loads add a third dimension where the mite can be free of viruses, virus-positive with replication or virus-positive without replication (asymptomatic). This combination of different states, morphotypes and virus loads produce a variety of acari which represents distinct targets with their own reachability.

In addition, the toxicity efficacy can be challenged by the ecological diversity of mites within colonies (Figure 2). In fact, there are four different states of *Varroa* females: (1) dispersal ecto-parasite attached to honeybee’s body, (2) comb mite freely moving inside the hive, (3) reproductive acari confined in brood cell and (4) non reproductive mite confined in brood cell [30,172]. This variety represents distinct targets with their own reachability, which has to be considered while studying molecule toxicity on *V. destructor*. Moreover, the mite population varies from winter to summer and seems adapted to each season. Females are smaller with larger shields and shorter legs in summer. The ratio of morphotypes is dynamic and goes from 20% of winter morphotype in summer to 20% of summer morphotype in winter [173,174]. Viruses loads add a third dimension where the mite can be free of viruses, positive with replication of the virus or positive without replication of the virus (asymptomatic).

Why extermination is not the solution. Treatments that eradicate susceptible acari keep the least sensitive to reproduce, which leads the *V. destructor* population to become highly resistant over time. It is especially the case with this ecto-parasite because of the high level of inbreeding within colonies. This makes the fixation of resistant alleles happen quicker [48]. To limit this phenomenon, at least temporarily, a rotation in molecular targets should be adopted [175]. Moreover, acaricides used at their lowest effective dose reduce the amount of residues stored in wax or honey, thus moderate the speed in acaricide resistance [176].

As a recent paper by Colin et al., (2020) [177] rightly advocated, acaricides should be tested at sub-lethal level to assess their impact on reproduction, nutrition or even orientation. It is a pity that toxicological studies rely mainly on lethal dose to evaluate the efficiency of the product while eradication of *V. destructor* is not an ecological long-term solution. However, preventing them from reproduction or right orientation, as supported by Soroker et al., (2019) [81], would reduce the selective pressure in favour of the most resistant mites. Other works also explained that the use of natural substances could grant a low level of *V. destructor* infestation, keeping honeybee colonies alive [33]. An integrated pest management strategy should thus, combine all the above ideas. It is necessary to understand the diversity of the *V. destructor* population within one single hive. Long-term alternative methods should rely on safer compounds (e.g., no interference with the nervous system) in small quantities altering host preference or mite reproduction through biocontrol and IPM to preserve honeybees.

## 4. Biocontrol and IPM Strategies for *V. destructor* Management

Here we summarised the main roads of IPM and biocontrol for honeybees against the ecto-parasite explored by scientists.

### 4.1. Biotechnical Approach 

Often combined with organic molecules, a mechanical approach can be an alternative as well. The main goal is to perform total brood interruption, removal of drone brood, queen caging or trapping combs to decrease the pressure of *V. destructor* population on the colony [131,178,179]. In fact, these mechanical methods allow to artificially create a broodless period where mites have to be on adult honeybees, making them accessible to molecules. The removal of brood frames after a broodless period can also allow the trapping of an important number of reproductive mites. Unfortunately, according to the region of the world, brood interruption does not give the same results. It was the best solution to lower the ecto-parasite pressure on colonies in several countries in Europe but not in the USA where it affected their strength and survival [179,180,181,182,183]. Another method called sugar shake, used as a diagnosis method, reduces as well the number of mites on adult honeybees and lower the pressure on the colony without causing deep damages [184].

Regrettably, these techniques are tedious, difficult to execute at large scale and can lead to honey losses which is a problem for beekeepers. Their great advantage is to keep the highest quality and safety standard for honeybee products while reducing *V. destructor* pressure [185].

### 4.2. Natural Predators

Another biological alternative in treatments against *V. destructor* relies on natural predators. Different species were identified as potential candidates. 

First, pseudoscorpions can occasionally be observed in hives and feed upon the ecto-parasite without harming bees [186,187]. They are able to paralyse and kill the mite by injecting their venom from their pincers [188]. Different species were described all over the world like *Nesochernes gracilis* or *Chelifer cancroides.* Some seemed beneficial to bees and others not [189]. Recent efforts showed that pseudoscorpions can be mass-reared, making it easier to consider them a long-term solution against *V. destructor* [190]. Yet, a gap in knowledge still needs to be filled regarding their actual utilisation in field experiments.

Second, the predatory mite *Stratiolaelaps scimitus* is another potential candidate studied for biocontrol solution. Despite an effective ability to kill *V. destructor*, they also prove to prefer the eggs of the honeybee to the mite [191]. Moreover, in field experiments, an introduction in early and late fall did not lead to a decrease in the acari pressure upon colonies [192,193].

It is quite difficult to find the perfect natural predator for *V. destructor*. An efficient mite predator would have to, at least, consume the ecto-parasite eggs or larvae, thus entering a brood cell in tandem with an adult mite [194]. Plus, this organism should not harm the bees, regardless of the stage of development which makes the task truly challenging.

### 4.3. Microbiota

Microbiota is a highly dynamic field of research. For honeybees, worker guts from colonies parasitised with *V. destructor* hold a higher proportion of *Snodgrassella alvi* and a smaller of *Lactobacillus* spp., indicating a modification of their microbiota induced by the acari [195,196]. Besides, healthy larvae host a large population of *Enterobacteriaceae,* meanwhile the infested one owns a diversified microbiota similar to the ecto-parasite microbiome [197]. Therefore, the microbiota is a new opportunity to dig in to fight the ecto-parasite. Various strategies have been already experimented by several scientific groups. One of them supports the use of transgenic gut bacteria for biocontrol purposes. A key point to do so, relies on the adaptation of the gut microbiota through transfer of plasmids and trans-conjugation between bacterial strains, making it the best place to transfer genes [198]. Leonard et al. [199] engineered a symbiotic bacterium from honeybees’ gut, *S. alvi*, producing repeatedly dsRNA against essential genes for the acari and were successfully fed to the bees. Ecto-parasites fed from bees nourished with the engineered bacteria died faster than mites fed upon control bees. This elegant research work is a real breakthrough and shows how significant bacteria from honeybees gut could help in the battle against the mite. It also shows that combining several treatment methods (e.g., dsRNAi and microbiota), can be an efficient way of reducing parasite pressure, nicely filling integrative pest management perspectives. Using a different approach based on the study of honeybee’s cuticle microbiota, where bacteria already fit the micro-environment of the hive, Sacca et Lodesani [200] also obtained encouraging results. They isolated strains able to induce *V. destructor*’s death within 3 days after spraying, namely *Lactobacillus kunkeei*, *Bacillus thuringiensis* or *Bifidobacterium asteroides*.

Rather than targeting the mite, honeybee microbiota can also be used to improve health and resistance in the host. Probiotics were already considered to enhance the immune system against other threats to honeybees like American foulbrood or *Nosema ceranae* [201,202,203]. As the acari was spotted to disturb honeybees’ gut microbiota (dysbiosis), it appeared that probiotics, like Gram-positive bacteria *Lactobacillus* and *Bacillus* strains, brought beneficial impacts on colony health and seemed to reduce the incidence of the mite [195,204,205]. Bacteria communities appealed as well for their released metabolites. They were tested as treatment against the ecto-parasite. Lactic acid from *L. johnsonii* AJ5 induced the mite’s death when fed to bees. The mechanism implied in *V. destructor*’s mortality remains unknown and needs further confirmations [206,207]. 

### 4.4. Pathogens

Pathogens are another avenue of potential sustainable solutions against the acari. Entomopathogenic bacteria like *Bacillus thuringiensis* (Bt) are widely used as bioinsecticide agents in crops [208,209]. Bt is a Gram-positive procaryote, naturally present on insects’ corpses as well as on leaf surface [210]. It penetrates the host via ingestion and produces crystal (protein called Cry) and vegetative toxins. In vitro studies showed that Bt is present on *V. destructor* corpses and were extracted to test their pathogenicity on mites as well as honeybees. They reported that, after being treated with Bt for 24 h, *V. destructor* trembled, regurgitated, with intestinal inflammation (dysentery) and eventually died [211,212,213]. Short-term experiment showed no lethal effects among *A. mellifera* adults or larvae and possibly reduced vertical displacement [213,214] whereas chronic exposure to Bt induced precocious mortality for adults and larvae bees [215]. A lack of field studies does not allow to consider Bt as a resolutive method against the mite. Moreover, resistance and biosafety are key issues raised by Bt use. The advantage of Bt relies on its fast-action and host specificities which should limit adverse effects on non-targeted organisms [209]. Yet, a watchful care should be taken due to several resistances from lepidoptera and coleoptera species already spotted to transgenic plants which produce Bt proteins [216]. 

Besides entomopathogenic bacteria, a wide range of studies used entomopathogenic fungi since they kill acarine species [194,217,218]. Entomopathogenic fungi spread into their hosts via specialised spores called conidia. It takes 3 to 10 days to destroy the host by a lack of nutrient, water distress, toxins impact and mechanical disruption [219]. In the biocontrol area, *Metarhizium anisopliae* and *Beauveria bassiana* are the figurehead with thousands of research papers. The former was studied several times by Kanga et al. [220,221,222,223] They explored the effectiveness of the fungi against the mite through dust versus coated strips and determined that broodless periods are more favourable. *M. anisopliae* seems very persistent due to its presence 42 days after treatment. Field experiments in Texas and Florida showed encouraging results. The efficient formulation seemed tricky but dry conidia sprinkled held good results. Unfortunately, the fungus was also shown to kill honeybees [220,224]. Italian researchers shed light on sub-lethal behavioural impacts of the fungi (*M. anisopliae var. anisopliae* BIPESCO 5) on the mite that held a repellent effect from nurses carrying conidia [225]. They led as well a field experiment for 24 days which resulted in lower pressure of the acari, although honeybees losses were detected [226]. Besides, *V. destructor* infested-brood inoculated with the fungi showed a recovery in the expression of hymenoptaecin gene, involved in immunity, thus correcting the immunosuppression induced by the mite [227]. To date it is not crystal clear whereas *M. anisopliae* is safe for honeybees if applied intra hive even considering the improved results showed by Hans et al. [228] with a modified strain. Further experiments are still needed.

Regarding *B. bassiana,* it seems naturally present in hives, even in brood cells sometimes [229]. Meikle et al. [172,230,231] explored the effect of the fungi on honeybees colonies health while infested by *V. destructor* in field experiments and could not clearly reveal a high efficacy. They investigated the best formulation for an easy use, knowing that conidia germination is a key issue difficult to standardise. If sprayed, it seems to reduce the survival of worker Africanised bees [232]. 

The main drawback of fungi and bacteria, regardless the lack of data about long-term effects, relies on low specificity of their toxins and complication for them to colonise and survive in the ecosystem of the hive [172,207]. In addition, from a phylogenetic perspective, the honeybee and the ecto-parasite are relatively close, making it harder to reach a single one. Conversely, their way of action targets multiple receptors which is supposed to result in a lower and/or slower evolution in host resistance. 

### 4.5. Double-Stranded RNA Interference (dsRNAi)

DsRNAi is often mentioned as a recent potential alternative for *V. destructor* pest management [37,73,81,175]. In fact, dsRNA induces the degradation of RNA with similar sequences. Therefore, the job of dsRNAi is to momentarily silence targeted gene expression, preventing the protein from filling its function. *Varroa destructor* holds the molecular machinery to be sensitive to RNAi treatment, administered orally or via dsRNA injection. Campbell et al. [233] showed for the first time that glutathione S-transferase (GST), a key enzyme in the detoxification pathway, could be decreased through dsRNAi injection or submersion of *V. destructor*. A few years later, they also demonstrated that neural peptides could be targeted [94]. Garbian et al. [234] went further and led an elegant experiment demonstrating that dsRNA could be delivered to *V. destructor* by inserting it in honeybees’ food. Their work unlocked the delivery issue making it much easier to administer. Besides, odorant receptors as well as genes involved in survival or reproduction were discovered in the mite using RNAi methods [235,236]. Odorant receptor knockdown even led to the augmentation of vitellogenin in the acari, simulating the change from non-reproductive to reproductive mode. Although it seems to be a promising method, its long-term and potential risk of mutations or off-target effects are still unclear and largely debated.

### 4.6. Chemo-Disruption

This alternative pest management involves volatiles disturbance associated with key moments in *V. destructor* life cycle [59,237,238]. This method requires a full description and understanding of honeybees as well as the mite chemical communication in order to disturb targeted behaviours only. In addition, it is critical to note that many compounds hold different roles and significance according to the diverse hive contexts [59]. Nevertheless, several research attempted to master chemo-disruption, mostly to interfere with the mite’s mating behaviour. In vitro and semi-field tests used oleic acid, the sexual pheromone for the mite, but also the hygienic pheromone for honeybees. The aim was to saturate brood cells with the compound to confuse the male and reduce its successful attempts of copulation with suitable females. This technique allowed to reduce by 20% the number of spermatozoa carried by females [72,149]. Besides sexual confusion, host selection disruption was studied through electrophysiological assay and some compounds like cy{2,2} showed efficient results, which pushed the mite to pick a forager instead of a nurse bee, reducing the chance to find a new suitable larvae in brood cells [51]. Several semiochemicals, around 60, were identified over time by researchers that modify the mite behaviour [60,66,75,81] but rarely tested at relevant colony concentration [238]. The tricky part about chemo-disruption in hives is to reach mites inside combs and on honeybees’ bodies with an easy application of molecules at a relevant concentration to be effective. This is even more challenging as most of the molecules are also involved in bee pheromonal communication.

Owing to in vitro rearing methods developed lately, a better understanding of molecular pathways, and accurate communication channels used by the ecto-parasite was achieved. It is crucial to put them back into reality through a holistic approach gathering ecological, genetic and physiological factors.

## 5. From the Laboratory to the Field: How to Be Realistic?

**Figure 3:**
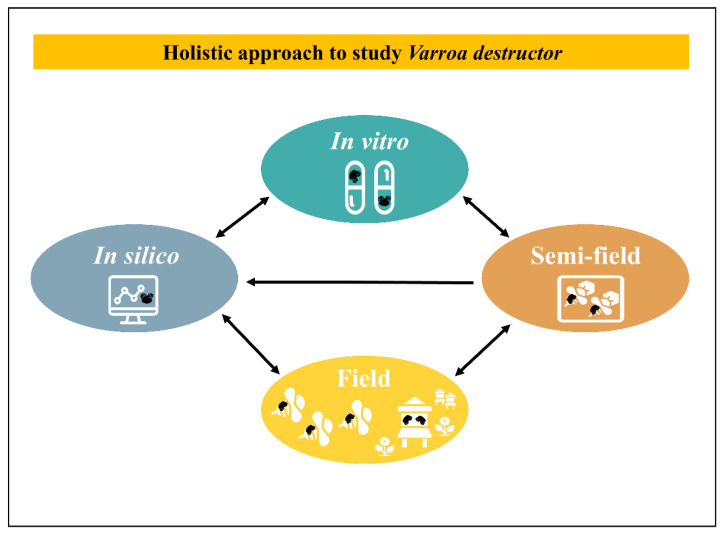
Holistic approach to study *V. destructor*. This process includes in silico, in vitro, semi-field, field tests and back to modelling with new enriched data. Each arrow indicates enriched exchanges between scales.

Clearly, to identify key factors in host–parasite relationships under laboratory conditions and to be able to transfer these conclusions into the field is a complex and laborious purpose. This is especially the case in studies about eusocial species with a completely different dynamic at the group level [239]. There is no common method to extrapolate laboratory results into the field. Yet, for each case a practical tactic could be examined with an integrative view. Theoretically, a holistic approach through an inverted funnel with a 4-steps loop and feedback could be conducted. This process includes in silico (modelling), in vitro rearing and testing, semi-field, field tests and back to modelling with new enriched data (Figure 3). A critical point to set up laboratory experiments is to find a trade-off between being close to natural conditions with complex but relevant conclusions and reducing the natural complexity to try to better interpret parameters of interest. To dig into that direction, accurate technical tools to study the relationship between the parasite and the host including their bacteria or viruses, are capital and sometimes still deficient. For example, the ultimate step for an in vitro rearing method relies on the development of a complete isolation of the ecto-parasite from the host. On the other side of the spectrum, field studies could benefit from easy to use technical tools to track honeybees on long distances [240]. Besides these technical issues and on a more applied research aspect many in vitro applications were often led but hardly pushed through the long run until a successful field product was released.

### 5.1. In Vitro Methods

To identify key elements in the physiology and biology of the mite with precise reproducibility, obtaining a large quantity of *V. destructor* is crucial. This is quite a challenge as relying on colonies implies high risks of colony losses and seasonal constraints [241]. In vitro rearing is thus, a promising tool for obtaining such a large number of mites while being able to study the parasite cycle in detail. The first main technical lock to study *V. destructor* is supported by its incapacity to survive or complete its cycle detached from its host and out of a brood cell, making an entirely controlled study complicated in the laboratory. That is why available in vitro rearing methods to study the reproductive acari are still dependent on the presence of bee larvae [183,242]. A full in vitro rearing with alive and reproductive daughters maintained out of the season is, to our knowledge, not available yet. Some current methods for instance use freshly collected larvae transferred into gelatine capsules, in which a foundress is inserted to obtain viable offspring after 12 days [243,244,245]. Maintenance of living *V. destructor* on synthetic feeding membranes is possible too. Parafilm or chitosan surfaces were created to study the nutrition, reproduction or virus infection of mites [25,36,246,247,248,249]. In these conditions, survival was possible for a few days and the initiation of reproduction was sometimes observed. In laboratory conditions, more than 20 molecules naturally emitted in bee colonies were identified but only a few were tested in field. For example, in natural conditions, after application of methyl palmitate to brood cells, Boot [250] did not observe any attractive effect like previously demonstrated in controlled environment [251]. This point shows how tricky it can be to transfer in vitro results into the field. 

While essential, those laboratory results still lack the countless sonar, tactile or olfactory cues naturally present within a hive. Semi field and field studies, on the other hand, allow the inclusion of such parameters to further understand the parasite cycle. 

### 5.2. Semi Field and Field Scales

SEMI-FIELD—An intermediate step between in hive and in vitro studies consists of semi-field experiments, for example by using beehive frames brought into the laboratory. Indeed, while being out of the full complex environment of the hive, it allows the inclusion of important sources of interactions, such as those emitted by the brood, the wax or the food. By considering part of this complex environment, Light et al. [252] showed that many of the *V. destructor* volatile attractants isolated in laboratory studies were not detected when a whole frame was analysed. Furthermore, in the case of chemo-disruption, if many kairomonal candidates have been identified [81], attempts of delivering these distracting odours in an accurate range in natural or semi natural conditions are scarce. To our knowledge, this crucial step from the laboratory to the field was only successfully achieved once in the case of sexual pheromone confusion [72]. One of the reasons for this void lies in the fact that establishing protocols to disturb the mite cycle without impacting honeybee communication is a critical task. 

FIELD—For eusocial species, like *A. mellifera*, studies at the colony level remain crucial. Unfortunately, in the darkness of the hive, to collect quantitative data on a wide range of individuals for long periods can be tricky. One of the solutions rely on connected hives [253]. It should allow us to measure some variables like temperature, hygrometry, vibrations but as well social exchanges for bees and why not mite movements. Some exciting new works caught inside videos of within comb cell exploring the movements of honeybees from egg to emergence as well as feeding behaviour from nurses to larvae [254,255]. This type of tracking is essential to reconnect laboratory observations to field experiments. The recent development and adaptation of gas sensor systems to beekeeping practices is really promising. They have already been used to infer a parasite pressure based on volatils detected in hives [256,257]. It is necessary to be able to precisely assess the state of the colony when infested with the acari, thus to use acaricides only if required and at the right moment [175,257]. From an IPM perspective and regardless of the agent, the formulation is also crucial. The active ingredient needs to survive in the environment of the hive and to access its target without or with the minimum off-target effects. The formulation of a biocontrol agent should thus depend on the environment where it is delivered. In the same way, the heterogeneity of the *V. destructor* population intra hive, between hives and locations has to be taken into account. The numerous sources of physiological, pathological, environmental, behavioural and genetic variations can interfere with the treatment against the mite and determine the success of the method (Figure 2) [172,174,258]. Several works took this aspect into account while testing different molecules or agents against the acari and showed a customised response per location [29,222,259]. To improve our knowledge about the host-parasite dynamic, we still need further research including these aspects to get the full picture from the molecular scale to the population dynamic [260]. 

### 5.3. In Silico (Modelling Approach)

Modelling approach is part of an integrative strategy to identify key elements in the host–parasite relationship using a high number of colonies. This powerful tool helps to represent complex systems, to test multi-factor hypotheses, to predict the outcome of host–pathogen interactions without the cost of individuals, time or money and to estimate crucial parameters from data [261,262,263]. Moreover, the use of models allows scientists to handle simulated experiments when conducting those experiments is realistically impossible. It is, thus a powerful tool to explore and understand the complexities of ecosystems [264], especially when associated with field data [265]. In the case of host-parasite dynamics, it is well-known that they are influenced by the exchange between parasite and host density. This phenomenon is illustrated through the central transmission-density function. Here the difficulty relies on the selection of the best model, the more representative of the reality [266]. However, it highlights crucial variables involved in the issue. For example, in the triangle *A. mellifera*, *V. destructor* and viruses like DWV or APV (acute paralysis virus) vectored by the mite, the modelling approach showed a similar population dynamic as the one measured in the field. It demonstrated that DWV reduces the number of healthy young bees accessing the overwintering population. This imbalanced situation in ages for the colony results in losses during winter or spring. Unfortunately, around 2000 *V. destructor* females in autumn seems enough to lead to death. The model predicted a widely spread DWV due to its lower virulence compared to another virus. They indicated as well that prior to the generation of overwintering honeybees, the ecto-parasite pressure should be reduced [267,268,269]. An interesting point with the latest development in modelling approach remains in the study of sub-lethal doses of pesticides and their combined effect on the colonies [270] as well as the long-term effect of antibiotics on bees [271]. Even acute contact toxicity was predicted recently [272], advocating for a deeper connection between in silico, in vitro, semi-field and field experiments. In the end, the host-parasite relationship should be part of an integrative view and inserted in a wider picture where other variables like poor nutrition, crop pesticides or pathogens are assessed to identify if they act in synergism or antagonism inside the hive [273].

## 6. Conclusions 

In the absence of a better solution, the current optimal strategy seems to be the use of oxalic acid in glycerol strips combined to biotechnical approach [32,179,181]. A tight monitoring of the infestation level is a key element to be effective. Rotation in time and space for treatments among hives can help to reduce the resistance spill over [48,274]. However, according to climate and country legislation the achievement and results can be dramatically different.

More than ever, researchers need to develop control strategies against *V. destructor* that adopt effective alternatives to hard chemicals, well-known to generate problems like residuals and resistance. If the use of organic acids, plant products and biological control were investigated these last years, none is conclusive for *V. destructor* management yet. The stumble point resides in the transfer of results obtained in the laboratory conditions to the field. In such a context, it would be necessary to intensify attempts, which rely on gene expression datasets, to discover a robust and reliable panel of health biomarkers towards integrated control of *V. destructor*. In the end, a holistic approach, evaluating at sub-lethal level different biotic and abiotic factors, will be a relevant pest management tool for sustainable apicultural and agricultural production while getting closer to field conditions.
